# The Genomic Analysis of Erythrocyte microRNA Expression in Sickle Cell Diseases

**DOI:** 10.1371/journal.pone.0002360

**Published:** 2008-06-04

**Authors:** Shao-Yin Chen, Yulei Wang, Marilyn J. Telen, Jen-Tsan Chi

**Affiliations:** 1 The Institute for Genome Sciences and Policy, Duke University School of Medicine, Durham, North Carolina, United States of America; 2 Department of Molecular Genetics and Microbiology, Duke University School of Medicine, Durham, North Carolina, United States of America; 3 Applied Biosystems, Foster City, California, United States of America; 4 Division of Hematology, Department of Medicine, Duke University School of Medicine, Durham, North Carolina, United States of America; East Carolina University, United States of America

## Abstract

**Background:**

Since mature erythrocytes are terminally differentiated cells without nuclei and organelles, it is commonly thought that they do not contain nucleic acids. In this study, we have re-examined this issue by analyzing the transcriptome of a purified population of human mature erythrocytes from individuals with normal hemoglobin (HbAA) and homozygous sickle cell disease (HbSS).

**Methods and Findings:**

Using a combination of microarray analysis, real-time RT-PCR and Northern blots, we found that mature erythrocytes, while lacking ribosomal and large-sized RNAs, contain abundant and diverse microRNAs. MicroRNA expression of erythrocytes was different from that of reticulocytes and leukocytes, and contributed the majority of the microRNA expression in whole blood. When we used microRNA microarrays to analyze erythrocytes from HbAA and HbSS individuals, we noted a dramatic difference in their microRNA expression pattern. We found that miR-320 played an important role for the down-regulation of its target gene, CD71 during reticulocyte terminal differentiation. Further investigation revealed that poor expression of miR-320 in HbSS cells was associated with their defective downregulation CD71 during terminal differentiation.

**Conclusions:**

In summary, we have discovered significant microRNA expression in human mature erythrocytes, which is dramatically altered in HbSS erythrocytes and their defect in terminal differentiation. Thus, the global analysis of microRNA expression in circulating erythrocytes can provide mechanistic insights into the disease phenotypes of erythrocyte diseases.

## Introduction

Erythrocytes constitute more than 90% of the cell population in the peripheral blood and are responsible for efficient gas exchange in the human body. Mature erythrocytes are the end-products of a highly regulated differentiation process that involves the gradual loss of cellular organelles, a decline in nucleic acid content, and a step-wise acquisition of erythrocyte characteristics [1]. One striking feature of erythroid differentiation is that the nuclei are extruded from cells as they differentiate into reticulocytes, the immediate precursor of mature erythrocytes. While cytoplasmic RNA and translation activities are still detectable in CD71+ reticulocytes, they fall below the detection limit as cells terminally differentiate to become CD71- mature erythrocytes [2]. Since hemoglobin makes up the majority of erythrocyte cellular proteins, these cells are frequently thought to serve merely as inert and passive containers of hemoglobins. However, their more dynamic nature was suggested by the recent finding that erythrocytes can mount cellular signaling and trigger functional responses during physiological and pathological stresses [3], [4]. This suggests that erythrocytes have a more sophisticated intracellular environment than previously appreciated.

Processes disrupting erythrocyte homeostasis lead to human diseases that present huge economic and social challenges. Even though erythrocyte diseases have been studied for a long time, our current understanding of the erythrocyte still cannot fully explain the multitude of clinical effects or the enormous clinical variation seen in patients with these diseases. Global analysis of gene expression using microarray technology holds great potential for advancing our understanding of erythrocyte diseases. This technology has led to an explosion of knowledge in understanding pathogenic mechanisms and clinical heterogeneity in human cancers. However, its application to erythrocyte diseases has been limited by the long-held belief that mature erythrocytes lack RNA expression. This belief is supported by the ability some RNA-binding dyes (such as thiazole orange or methylene blue) to stain reticulocytes but not erythrocytes, an observation which forms the basis of the clinical utility of these dyes in distinguishing reticulocytes from mature erythrocytes [5]. Furthermore, analysis of RNA isolated from whole blood by PAXgene technology [6] revealed no detectable contribution from erythrocytes. Only gene signatures from neutrophils, lymphocytes and reticulocytes were detected, even though erythrocytes are the predominant cell type in the blood [7].

Given the potential limitations of sensitivity and size bias for these traditional means of isolating and characterizing erythrocyte RNA, it remained possible that erythrocytes may contain RNA species not previously identified. Here, we provide several lines of evidence that human mature erythrocytes, although lacking in ribosomal and large-sized RNAs, contain diverse and abundant microRNAs. Our findings confirm the results of a recent study on small RNA expression in *Plasmodium falciparum* that found several microRNA species in peripheral erythrocytes [8]. Importantly, the discovery of these microRNAs allowed us to use microarrays to compare their expression in mature erythrocytes from normal (HbAA) and homozygous sickle cell (HbSS) individuals and to identify a link between the dysregulation of microRNA and the pathogenesis of sickle cell disease (SCD).

## Results

### Purification of Human Mature Erythrocytes

**Figure 1:**
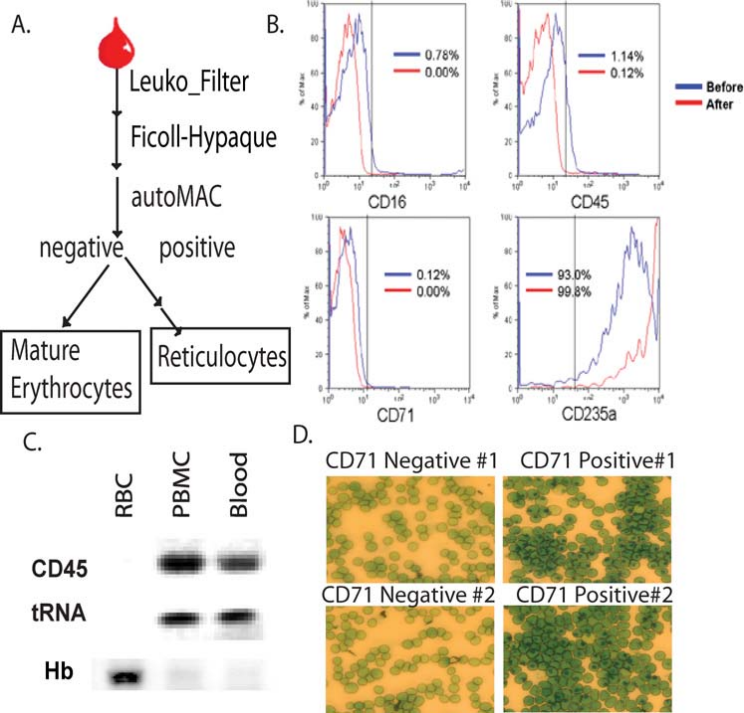
The purification of human mature erythrocytes. (A) The purification scheme used to obtain mature erythrocytes and reticulocytes from human blood. Assessment of the purified mature erythrocytes was then performed by examining the surface expression of indicated lineage markers on whole blood (before purification–blue line) and purified erythrocytes (after purification–red line) with FACS–CD16, CD45, CD71 and CD235a (B). (C) RT-PCR assay to evaluate the abundance of indicated transcripts (CD45, tRNA and beta-hemoglobin) in the RNA from purified erythrocytes (RBC), peripheral blood mononuclear cells (PBMC) and whole blood. (D) New methylene blue stain of the CD71- erythrocytes (left) and CD71+ reticulocytes (right) from two independent isolations.

To test for the possibility that mature erythrocytes contain previously undetected RNAs, we developed a protocol to obtain a pure population of mature erythrocytes by removing other blood cells through a series of purification procedures (Fig 1A). Blood collected from healthy volunteers first went through a PALL PurecellNeo filter to remove most leukocytes and was then placed in a Ficoll-hypaque density gradient to separate erythrocytes from leukocytes and platelets. Finally, any remaining leukocytes (CD45+) and reticulocytes (CD71+) in the packed erythrocyte pellets were removed by magnetic immuno-depletion with AutoMacs.

The purity of the erythrocytes obtained by this method was assessed with lineage-specific markers. Purified cells were 99.8% positive for the erythrocyte marker CD235a, while negative for leukocyte (CD45, CD16) and reticulocyte (CD71) markers (Fig 1B). These results correlated well with molecular evidence for leukocyte depletion, in that no CD45 mRNA was detected by RT-PCR (Fig 1C). The absence of reticulocytes in our erythrocyte preparations was validated by the absence of positive cells when stained with new methylene blue (Fig 1D) as well as routine reticulocyte analysis in the clinical laboratory. Taken together, these results show that we were able to obtain highly purified mature erythrocytes without significant contamination of leukocytes and reticulocytes.

### Identification and genomic characterization of erythrocyte microRNAs

To ensure that RNAs of all sizes were recovered from erythrocytes, we chose an RNA purification method designed for microRNA isolation, capable of capturing RNAs as small as ten nucleotides (nt). By this means of RNA purification, we obtained low but reproducible levels of RNAs from all mature erythrocyte samples (N = 21). The RNA yields ranged from 5–10 µg RNAs from 10 ml of whole blood; by back-calculation, considering the cell numbers and cell purification yield, with the assumption of equal RNAs in all erythrocytes, the RNA content was approximately 2–3×10^−4^ pg/cell. This RNA content is similar to platelets (3×10^−4^ pg/cell), but significantly lower than nucleated cells (5–9 pg/cell) [9]. To rule out the possibility that the RNAs were from contaminating reticulocytes, we adopted an alternative approach to separate reticulocytes from erythrocytes with an arabinogalactan density gradient [10]. With this independent approach of isolating mature erythrocytes, we obtained a similar level of erythrocyte RNA.

**Figure 2:**
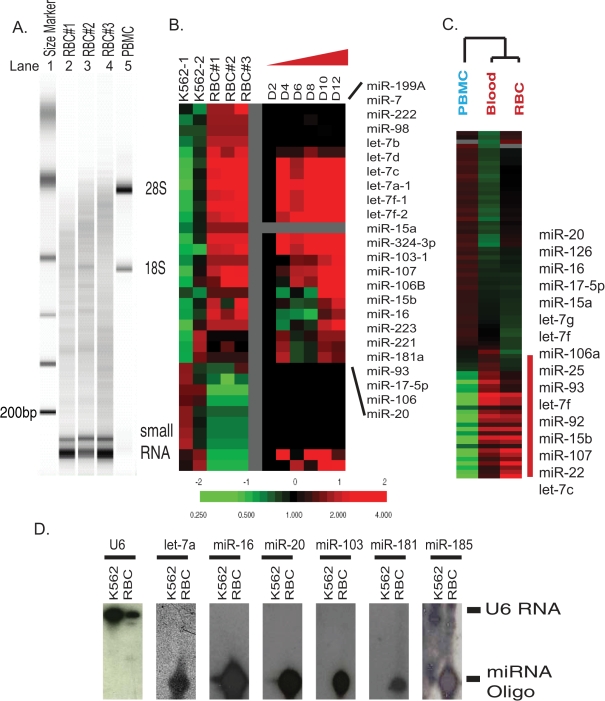
Human mature erythrocytes contain abundant and diverse microRNAs. (A) The size distribution of RNAs of three independent erythrocyte samples (lanes 2–4 labeled RBC #1,2,3) and one PBMC sample (lane 5 labeled as PBMC) were assessed with Agilent Bioanalyzer with indicated size markers (lane 1 as indicated). (B) Left: the microRNA expression pattern obtained with microRNA microarrays of three mature erythrocyte samples was compared to that of two erythroleukemia K562 cell lines. Right: the expression of erythrocyte-specific microRNAs in the CD34+ erythroid progenitor cells at indicated stages of erythroid differentiation in a previously published study [14]. (C) The microRNA expression of whole blood was similar to erythrocytes and grouped together in one branch away from leukocytes with unsupervised hierarchical clustering. Selected erythrocyte-specific microRNAs were shown. (D) The expression of U6 and indicated microRNAs in K562 cells and erythrocytes was assessed by Northern blots with the migration position of U6 and mature microRNAs indicated. The position of U6 RNA and microRNAs (indicated bars on the right) were established with synthesized DNA oligonucleotides included in the Northern blots.

We then used an Agilent bioanalyzer to compare the size distribution of erythrocyte RNAs with leukocyte RNAs (Fig 2A). Erythrocyte RNAs (Fig 2A, lane 2–4) did not contain the two distinct ribosomal bands (28S and 18S) observed in simultaneously prepared peripheral blood mononuclear cells (PBMC) (Fig 2A, lane 5). Instead, erythrocyte RNAs was highly enriched for small-sized RNAs of less than 200 base pairs, which were also present in leukocyte RNAs but at a much lower level. These small-sized RNAs were sensitive to treatment with RNAse and NaOH, while resistant to DNAse treatment. The observed size range of erythrocyte RNAs could represent several RNA species, including microRNA, snoRNA, tRNA and degraded mRNAs. MicroRNAs are evolutionarily conserved, small, non-coding RNA molecules that play important regulatory roles in various biological processes [11]. To assess the possibility that the small-sized RNAs contained microRNAs, we utilized a spotted microRNA microarray platform containing DNA oligonucleotides representing 196 human microRNAs [12] to interrogate the microRNA contents of mature erythrocytes and K562, an erythroleukemia cell line which expresses many genes typical of an earlier stage of erythroid differentiation. Equal amounts (40 ng) of fractionated RNAs from three separately purified erythrocyte and two K562 samples were labeled for microarray analysis. As shown in Fig 2A, the hybridization result revealed abundant and diverse microRNAs within the erythrocyte RNAs and 87 microRNAs whose expression varied between erythrocyte and K562 more than 3 fold in at least two samples. When compared with K562 cells, mature erythrocytes specifically expressed several microRNAs, including several microRNAs in the let-7 family and several microRNAs (miR-181A, miR-223, miR-15, miR-16) that have been shown to play a role in the lineage differentiation in the hematopoietic system [13].

To examine changes in microRNA expression levels during the process of erythroid differentiation, we compared our array results with microRNA expression in the differentiating hematopoietic CD34+ cells described in the supplemental data of a study on cancer microRNA expression [14]. Even though the CD34+ cells did not reach the stage of reticulocytes at the end of this *in vitro* differentiation process, most of the erythrocyte-specific microRNAs found in our array analysis were upregulated during the early stages of erythroid differentiation (Fig 2B). Similar sets of microRNAs were also found in two other studies of microRNA expression with different *in vitro* erythroid differentiation models [15], [16]. Despite the fact that erythrocytes are the predominant cell type in the peripheral blood, the messenger RNA (mRNA) from leukocytes contributes the majority of mRNA in whole blood, since these nucleated cells have much higher RNA content [7]. Our finding of microRNA expression in erythrocytes prompted us to test whether erythrocyte microRNA may contribute significantly to the microRNA expression pattern seen in whole blood. To test this possibility, RNAs from whole blood, erythrocytes, and leukocytes from the same healthy normal individuals (N = 3) were fractionated and an equal amount of small-sized RNAs (<40 bp) was labeled and interrogated with microRNA microarrays. The unsupervised hierarchical clustering of these samples consistently arranged whole blood samples and erythrocyte samples in the same branch away from the leukocyte samples. This showed that the whole blood microRNA expression pattern was similar to that of erythrocytes and not that of leukocytes (Fig. 2C). This observation is distinct from the results obtained with mRNA and further highlights the important contribution of erythrocyte microRNA to whole blood microRNA expression. Therefore, leukocyte microRNAs represent a relatively minor portion of whole blood microRNAs and are not likely to be the source of the microRNAs we have discovered in mature erythrocytes. The erythrocyte-specific microRNA gene cluster found when compared with leukocytes (Fig 2C) contained the same sets of erythrocyte-specific microRNAs seen when erythrocytes were compared with K562 in the previous analysis (Fig 2B). We chose six of erythrocyte-specific microRNAs and further validated their abundant expression in erythrocytes by Northern blot (Fig 2D). All of the tested microRNAs were present predominantly in their mature form in the erythrocytes. No significant precursor microRNAs or microRNA degradation products were noted in the Northern blots.

**Figure 3:**
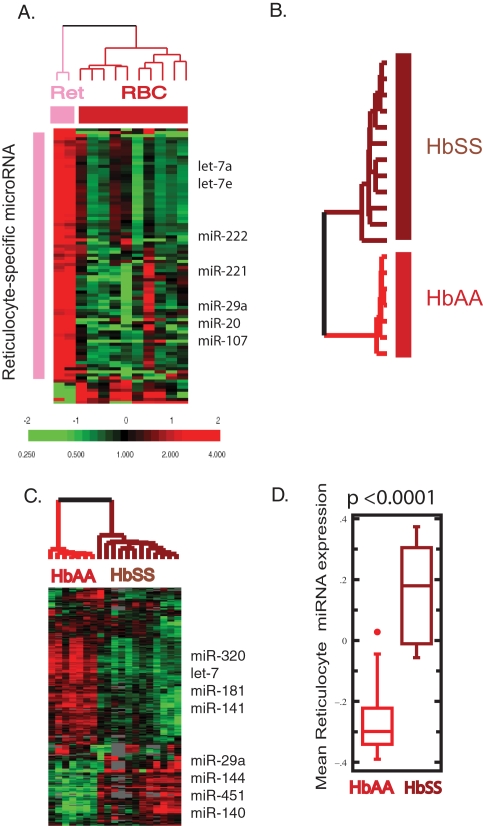
Erythrocyte microRNA show differentiation- and disease-specific expression. (A) The expression value of microRNA expression in the two reticulocytes (pink) and ten erythrocytes (red) assessed with TaqMan real-time assay. The expression levels of the 83 microRNAs identified by SAM as reticulocyte-specific were shown with selected microRNAs indicated. (B, C) The erythrocyte microRNA expression obtained from 7 HbAA (red) vs. 12 HbSS (brown) samples assessed with microRNA microarrays are distinct and grouped separately with unsupervised hierarchical clustering with several differentially expressed microRNAs indicated. (D) The average expression value of the 83 reticulocyte-specific microRNAs in the HbSS (brown) erythrocytes was significantly higher than that of HbAA (red) erythrocytes.

Recently, a stem-loop real-time PCR technology has been developed to determine microRNA expression levels, with a large dynamic range [17] in minute amounts of samples [18]. Importantly, this technology relies on the specific finding of both the 5′ and 3′ ends of microRNA and therefore detects only full length mature microRNAs but not their precursors or partially degraded products. We used this real-time RT-PCR assay to determine the expression level of 192 mature microRNAs (for which the assay was available) in 10 erythrocyte and 2 reticulocyte samples to compare the microRNA expression for erythroid cells at these two different differentiation stages. The reticulocytes were obtained by additional purification with cell sorting using FACS from the CD71+ fraction obtained during the process of mature erythrocyte purification, based on CD71 immunodepletion (Fig 1A). Out of 192 assayed microRNAs, 162 were highly detectable (with cycle threshold (CT) <35) in more than 75% of the samples and were chosen for further analysis. This result indicated that the reticulocytes and erythrocytes possess abundant and diverse groups of microRNAs, which were full length mature microRNAs instead of partially degraded microRNA remnants. Out of these 162 microRNAs, which were expressed in erythrocytes, 136 of them were represented on our microRNA microarrays and 130 exhibited hybridization signals in the Cy5 channel more than 2 fold above background. MicroRNAs in the let-7 family were among the most abundant transcripts in real-time PCR assays, consistent with our results from microarrays. We normalized the expression levels of the 162 microRNAs against miR-152 expression levels in all 12 samples for further analysis. We used miR-152 as an endogenous normalization control, since its expression was not altered during different stages of erythroid differentiation [14], and it showed the least amount of variation across 40 different human tissues in a separate microRNA real-time RT-PCR profiling study (Applied Biosystems, unpublished data). The resulting negative normalized Ct value (as represented by −ΔCt*_i_* = −(Ct*_i_*−Ct_miR152_)) of all remaining 161 microRNAs indicated the expression level of each microRNA (detailed in supplemental website). Unsupervised hierarchical clustering based on the expression values grouped all samples into two separate branches–one branch containing all freshly isolated erythrocytes and another branch containing reticulocytes (displayed as a heat map in Fig 3A). Therefore, mature erythrocytes have a microRNA expression pattern distinct from that of reticulocytes. We have further applied Significant Analysis of Microarrays (SAM) [19] in a supervised analysis and found that 83 microRNAs were expressed in a reticulocyte-enriched fashion (Fig 3A and Table 1). These reticulocyte-enriched microRNAs included microRNAs of the let-7 family as well as miR-221 and miR-222, two microRNAs whose downregulation during erythroid differentiation is essential this process [20].

**Table 1:** The 83 microRNAs with preferential expression in reticulocytes.

Reticulocyte-specific microRNAs
hsa-miR-125b
hsa-miR-321
hsa-miR-193
hsa-miR-145
hsa-miR-128b
hsa-miR-221
hsa-let-7f
hsa-miR-99b
hsa-let-7a
hsa-let-7g
hsa-miR-19a
hsa-miR-122a
hsa-miR-146
hsa-miR-129
hsa-miR-151
hsa-miR-373
hsa-miR-188
hsa-miR-197
hsa-miR-187
hsa-let-7d
hsa-miR-339
hsa-let-7c
hsa-let-7e
hsa-miR-338
hsa-miR-143
hsa-let-7b
hsa-miR-196b
hsa-miR-370
hsa-miR-93
hsa-miR-345
hsa-miR-103
hsa-miR-125a
hsa-miR-302a*
hsa-mir-196-1
hsa-miR-198
hsa-miR-216
hsa-miR-199a
hsa-miR-139
hsa-miR-196
hsa-miR-373*
hsa-miR-330
hsa-miR-337
hsa-miR-10b
hsa-miR-324-3p
hsa-miR-222
hsa-miR-184
hsa-let-7i
hsa-miR-189
hsa-miR-26b
hsa-miR-196a
hsa-miR-19b
hsa-miR-342
hsa-miR-135a
hsa-miR-199a*
hsa-miR-200a
hsa-miR-105
hsa-miR-210
hsa-miR-107
hsa-miR-147
hsa-miR-20
hsa-miR-325
hsa-miR-98
hsa-miR-138
hsa-miR-194
hsa-miR-218
hsa-miR-23b
hsa-miR-135b
hsa-miR-137
hsa-miR-108
hsa-miR-15b
hsa-miR-211
hsa-miR-340
hsa-miR-220
hsa-mir-124b
hsa-miR-195
hsa-miR-185
hsa-miR-29a
hsa-miR-302b*
hsa-miR-95
hsa-miR-223
hsa-miR-101
hsa-miR-30c
hsa-miR-27a

To determine whether the global analysis erythrocyte microRNA expression can lead to novel insights in human erythrocyte diseases, we recruited 12 individuals with HbSS (homozygous sickle cell disease) and 7 race-matched individuals with HbAA (normal hemoglobin). We used the three-step purification method based on both density separation and immuno-depletion of the reticulocyte population (Fig 1A) to isolate erythrocytes for RNA analysis since the density-only approach may suffer potential reticulocyte contamination in “dense” cell fractions from the HbSS patients. Using FACS, methylene blue stain and clinical laboratory techniques we confirmed that the purification scheme would allow us to obtain a mature erythrocyte population from HbSS individuals, who typically have higher levels of reticulocytes than normal individuals. The microRNA expression pattern of the purified erythrocytes from these 19 individuals was determined by microRNA microarrays. The microRNA expression patterns in HbSS and HbAA erythrocytes were distinct from each other and were easily separated into two groups by unsupervised hierarchical clustering (Fig. 3B) based on the 200 array elements (97 microRNAs) varying by more than 1.8 fold from the mean in two samples. Several microRNAs (miR-320, let-7s, miR-181, miR-141) were over-represented in the HbAA erythrocytes, while other microRNAs (miR-29a, miR-144, miR-451, miR-140) were over-represented in the HbSS erythrocytes (Fig. 3C). To compare the differentiation status of the two sample groups, we extracted expression values of the 83 reticulocyte-enriched microRNAs (Fig 3A) and found that their expression level was significantly higher in HbSS than in HbAA erythrocytes (Fig 3D, p<0.0001). This result suggested that HbSS erythrocytes exhibited an expression pattern closer to reticulocytes, consistent with their relatively younger age due to their shorter life span (∼20 vs. 120 days) [1]. There were also many other differences in the microRNA expression patterns of HbSS and HbAA erythrocytes that may not related to differentiation stages or erythrocyte ages. The basis for these differences in microRNA expression is currently unknown.

### miR-320 dysregulation and CD71 phenotypes in HbSS individuals

**Figure 4:**
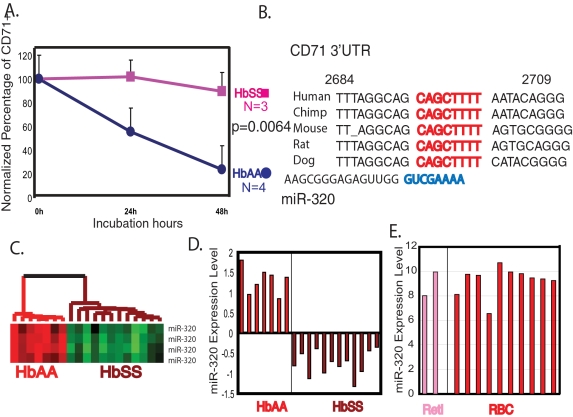
CD71 downregulation during terminal differentiation is defective in HbSS reticulocytes and is a potential mRNA candidate for miR-320 regulation. (A) HbSS reticulocytes exhibited defective terminal differentiation. Four purified HbAA (red) and three purified HbSS (brown) reticulocyte samples were placed in differentiation culture media containing autologous plasma. The percentage of remaining CD71+ cells (normalized against the starting CD71+ percentage) after 24 and 48 hours was significantly higher for HbSS samples than HbAA samples (p = 0.0064). (B) The target sequence (red) of the CD71 3′UTR of the five indicated species was aligned with the seed sequence (blue) of miR-320. (C, D) The relative expression of miR-320 among the HbAA vs. HbSS erythrocytes is shown as heat map (C) or bar graph of centered mean expression values (D). (E) The relative expression of miR-320 in two reticulocyte (pink) and ten erythrocytes (red) samples was assessed with real-time PCR and shown.

Although the high number of CD71 positive reticulotyces (reticulocytosis) seen in HbSS individuals is thought to reflect increased erythropoiesis in response to hemolysis, we questioned whether there might also be a defect in terminal differentiation in HbSS reticulocytes for which microRNAs play a role. CD71 (transferrin receptor) is important both in the capturing of transferrin/iron complex and the most relevant differentiation marker for the terminal differentiation. Given the importance of RNA-binding proteins in the regulation of CD71 expression at the post-transcriptional level where microRNAs typically function [21]. To test this possibility, we placed purified CD71+ reticulocytes in a previously described *in vitro* differentiation model [22]. Reticulocytes from all four healthy donors underwent successful terminal differentiation within 48 hours, as evidenced by loss of surface expression of CD71, disappearance of new methylene blue staining, decrease in mean corpuscular volume (MCV) as reflected in the increase in mean corpuscular hemoglobin concentration (MCHC) (Fig 4A and Fig S1) [22], [23]. In contrast, reticulocytes from all three tested HbSS individuals failed to manifest these changes of terminal differentiation; they had persistent surface expression of CD71 (p = 0.0064) and no changes in MCHC during the 48 hours of incubation (Fig 4A and Fig S1). This result indicated there was a defect in terminal differentiation in the HbSS reticulocytes and was consistent with our observation that HbSS erythrocytes appeared to have a less differentiated phenotypes.

To evaluate the potential role for dysregulated microRNAs in the defective downregulation of CD71 in HbSS reticulocytes, we used several predictive algorithms to identify the microRNAs likely to regulate the 3′UTR of CD71 [24]–[26]. MiR-320 was predicted to regulate CD71 by all three predictive algorithms (TargetScans [24], PicTar [25] and miRanda [26]) with a perfect match between its “seed” sequence (5′- GUCGAAAA-3′, nucleotides 2–8) and the regulatory region in the CD71 3′ UTR (CAGCTTTT, 2693 to 2700 of CD71 mRNA) that is conserved in five species (Fig 4B). Furthermore, the miR-320 expression level, which was very high in HbAA erythrocytes, was dramatically reduced in HbSS erythrocytes (Fig 4C, D), consistent with a possible role in the defective repression of CD71 seen in HbSS cells. The miR-320 expression level did not change significantly between reticulocyte and erythrocyte samples in our real-time RT-PCR analysis of HbAA cells (Fig 4E).

**Figure 5:**
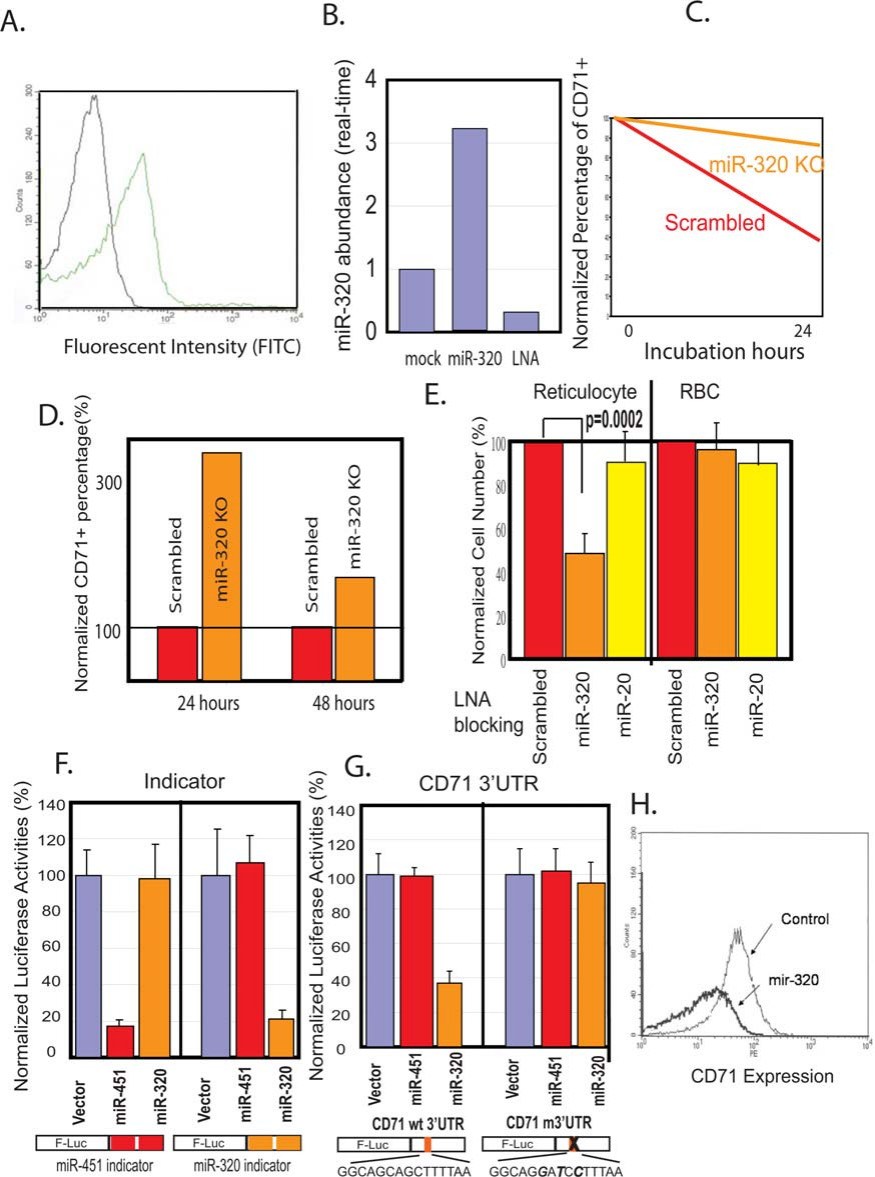
Poor expression of miR-320 in HbSS cells was associated with defective CD71 downregulation during terminal differentiation. (A) The histogram of the fluorescent intensity of reticulocytes transfected with unlabeled (black line) or fluorescently-labeled miR-320 (green line). (B) The miR-320 level in the transfected reticulocytes transfected with mock, synthesized miR-320 or LNA targeting miR-320 was determined with real-time PCR. (C, D) The percentage of remaining CD71+ cells normalized against the initial percentage after *in vitro* differentiation at indicated time points after transfection with either scrambled LNA or miR-320 knock down LNA. (E) Cell numbers of reticulocytes (left) and erythrocytes (right) 24 hours after transfection with treatments blocking indicated microRNAs were shown. (F). The reporter activities when empty vector or miR-451 and miR-320 expression constructs were co-transfected into K562 cells with respective indicator plasmids containing duplicated copies of sequences identical to miR-451 and mIR-320. (G) The over-expression of miR-320, but not miR-451 or empty vector, in K562 can suppress a reporter construct containing the 3′UTR of CD71. This suppression was abolished when three nucleotides in the miR-320 predicted target site were mutated as indicated in the 3′UTR of CD71. (H) Overexpression of miR-320 led to lower levels of CD71 in K562 cells 48 hours after transfection.

To directly test the functional role of miR-320 during terminal differentiation, we developed a transfection technique that allowed us to elevate and reduce the levels of selected microRNAs in reticulocytes. Using a modified protocol with fluorescently labeled oligonucleotides and lipofectamine, we achieved a transfection efficiency of 40–50% in both erythrocytes and reticulocytes (Fig 5A). When synthesized mature miR-320 was transfected into reticulocytes, we detected a three-fold increase in miR-320 expression with real-time RT-PCR (Fig 5B). On the other hand, the transfection of LNA antisense oligonucleotides against miR-320 led to 80% reduction in miR-320 levels (Fig 5B). To investigate the function of miR-320 during reticulocyte terminal differentiation, we inhibited miR-320 function by transfecting either LNA antisense oligonucleotides against miR-320 or a scrambled sequence LNA oligonucleotide into HbAA reticulocytes before culturing them for *in vitro* terminal differentiation. While reticulocytes transfected with the scrambled sequence LNA oligonucleotide underwent normal CD71 downregulation, miR-320 inhibition by the antisense oligonucelotide led to a persistent high expression of CD71 (Fig 5C, D) in three separate experiments. In addition, miR-320 inhibition in reticulocytes caused significant cell death and led to fewer cell numbers when compared with cells transfected with the scrambled oligonucleotide or with miR-20a antisense oligonucleotides (Fig 5E, left). The effect was stage-specific, since mature erythrocytes were not sensitive to the same treatment (Fig 5E). These results indicated an essential role for miR-320 in maintaining erythrocyte homeostasis during terminal differentiation in normal reticulocytes. The phenotype of miR-320 inhibited normal cells resembled that of HbSS cells, including defective maturation and decreased survival. This suggested that low miR-320 expression may have a role in the dysregulated maturation and decreased cell survival seen in SCD [1].

To evaluate whether CD71 was a direct target of miR-320, we generated CMV-based expression constructs containing the miR-451 and miR-320 genomic sequences and then tested for their ability to specifically suppress expression from their respective “indicator” reporter constructs containing two copies of identical miR-451 or miR-320 target sequences in a specific manner (Fig 5F). A 453bp region of the CD71 3′UTR (2547–2999 bp of CD71 mRNA) was then amplified and placed downstream of the luciferase reporter gene (Fig 5G). When this reporter construct was co-transfected into K562 cells with expression constructs encoding miR-320, miR-451 or empty vectors, we found that only miR-320, but not miR-451 or empty vectors, repressed its activity (Fig 5G, left). This miR-320-mediated repression was dependent on the predicted miR-320 target site in the CD71 3′UTR since a three base pair change in this site abolished miR-320-mediated repression (Fig 5G, right). Collectively, these results indicated that CD71 was a direct target of miR-320 via the interaction between its 3′UTR and the miR-320 seed sequence.

To further validate the miR-320/CD71 interaction, we overexpressed miR-320 in K562 cells and examine their influence on surface CD71 expression. Overexpression of miR-320 in K562 cells led to a significantly lower level of CD71 when compared with control transfection with empty vectors (Fig 5H). Taken together, these results showed that miR-320 can directly regulate the expression of CD71 and suggested that the poor expression of miR-320 in HbSS cells is associated with their persistently high CD71 level during terminal differentiation. In addition to CD71, miR-320 was also predicted by TargetScanS [24] and PicTar to target other mRNAs (Table S1, S2 [1]), including ETS2 (v-ets erythroblastosis virus E26 oncogene homolog 2) and EPB41L5 (erythrocyte membrane protein band 4.1 like 5), two mRNA encoding proteins important for erythrocyte biology

## Discussion

In this study, we have presented several lines of evidences for the presence of diverse microRNA in the mature erythrocytes. In gene expression studies of enucleated cells with low RNA content (such as erythrocytes, reticulocytes and platelets) [2], [9], [27], [28], one important concern is that a minority of contaminating nucleated cells with high RNA content (leukocytes) may distort the observed gene expression patterns. We do not believe that such contamination has significantly affected this study, because of the following observations. First, we have employed two different purification schemes, composed of three levels of cell separation based on cell density and surface expression to remove most leukocytes, platelets and reticulocytes, to obtain the mature erythrocytes used for expression analysis. We confirmed the purity of the resulting mature erythrocytes by FACS, methylene blue staining and molecular analysis of isolated RNAs. Second, the size distribution and characteristics of the isolated erythrocyte RNAs were significantly different from that of all nucleated cells–uniquely enriched for small RNA species without prominent bands of ribosomal RNAs. Third, when microRNAs from whole blood were compared with leukocytes and mature erythrocytes, the expression pattern of whole blood, even without leukocyte depletion, was derived mainly from erythrocytes instead of leukocytes. The microRNA expression pattern of mature erythrocytes was also different from reticulocytes using microRNA real-time RT-PCR assays. Taken together, these results indicate that the expression of these microRNAs is indeed from mature erythrocytes and not from other “contaminating” cells. Knowledge of erythrocyte microRNA is also important to investigators who are interested in leukocyte microRNAs, as it is now clear that they must avoid the significant and unexpected bias caused by erythrocyte microRNAs.

The human erythrocyte is one of the most studied cell types and is often used as a model system to understand the general principles of molecular genetics, biochemistry, membrane biology and cell physiology. However, our understanding of erythrocytes and the diseases that affect them is still incomplete. The unexpected discovery of microRNAs in erythrocytes, consistent with a previous study of parasitized red cells [8], adds a new dimension to erythrocyte characterization and has the potential to enhance our understanding of their phenotypic alterations during physiological and pathological adaptations. As a step toward realizing this potential, we used microarrays to analyze erythrocyte microRNA gene expression in SCD to establish a connection between microRNA dysregulation and the certain disease phenotypes of SCD. Several recent studies have analyzed microRNA expression on a global scale during *in vitro* erythroid differentiation [15], [16], [29]. Although these differentiation models do not lead to terminally differentiated erythrocytes analyzed in our study, we note a high degree of similarity in the microRNAs seen during the earlier stages of erythroid differentiation investigated in these studies and the microRNAs reported here in mature erythrocytes. This similarity indicates that the microRNAs present in mature erythrocytes are likely to originate from cells in earlier differentiation stages prior to nuclear exclusion and persist in the mature erythrocyte after terminal differentiation. The selective presence of these microRNAs in these post-mitotic erythrocytes may be due to the longer half-life and slower decay kinetics of specific microRNAs. Our results indicated that microRNAs still exist in the functional form in reticulocytes, consistent with previous study [30]. The immediate precursor of reticulocytes, orthochromatic normoblasts, still contains nuclei. Since the reticulocyte stage lasts for 40 hours [1], it is conceivable that the reticulocyte microRNA synthesized in the orthochromatic normoblast become processed, fed into the maturation pathways and persist in a functional form in reticulocytes. These microRNAs may persist in the mature erythrocytes after the terminal differentiation.

The main functional mechanism of microRNAs is through the post-transcriptional regulation of their target mRNA via mRNA degradation or translation inhibition [11]. What are the potential roles for microRNAs in mature erythrocytes, a cell type with limited target mRNA and translation activity? What is the clinical relevance for genomic analysis of erythrocyte microRNAs? First, these erythrocyte microRNAs are likely to play important regulatory roles during the earlier stages of erythropoiesis, as late as the reticulocyte. Translational activities are still present in the reticulocyte stage, and our results also clearly demonstrate a functional role of microRNAs in these cells during terminal differentiation of reticulocytes. This observation are consistent with a recent study of recapitulating the microRNA-mediated translation repression in reticulocyte cell lysates [30]. Thus, the microRNA composition captured in circulating erythrocytes is the balance between microRNA accumulation from earlier differentiation stages and the subsequent microRNA decay in the remaining erythrocytes exposed to various physiological and pathological conditions. Many other studies have highlighted the functional importance of several microRNAs (e.g. miR-221, -222, -451 and -24) during various stages of erythroid differentiation [16], [20], [31]. Second, erythrocyte microRNAs may play a direct role in the de-adenylation and degradation of mRNA during erythrocyte terminal differentiation as suggested by several studies [32]–[34]. Third, it is possible that microRNAs play a role in the host-pathogen interaction between erythrocyte and malaria parasites, given that human microRNAs are found in the malaria parasites *P. falciparum* [8]. Similar involvement of microRNAs in the host-pathogen interaction was shown for host microRNAs affecting the growth and propagation of pathogen [35], [36] as well as pathogen-encoded microRNAs impacting host cellular physiology [37]–[39]. In our analysis, HbSS erythrocytes have a very high level of miR-451, which was found be translocated into *P. falciparum.* This observation leads to the possibility that microRNA composition may contribute to the malaria resistance notes for sickle cell erythrocytes [40], [41]. Finally, microRNAs may have additional functional roles in erythrocytes via a mechanism independent of mRNA targeting as suggested in our observation that the blockage of mIR-320 leads to decreased erythrocyte survival.

The differential expression of erythrocyte microRNA may also lead to novel insights into human erythrocyte diseases. We observed a dramatic and significant difference between microRNA expressions in mature HbSS and HbAA erythrocytes; partly because of HbSS erythrocytes are younger than HbAA erythrocytes in their microRNA expression pattern, consistent with their shorter life span. These differences are likely to provide additional information about the disease phenotypes, similar to the miR-320::CD71 connection established in our current study. Linkage of particular microRNAs to particular SCD disease phenotypes offers the potential not only to develop useful biomarkers as suggested in a recent review [42], but also to generate testable biological hypotheses and relevant pathological insights based on these microRNAs and their predicted target mRNAs. This line of research can lead to an enhanced understanding of the relevant pathophysiological mechanisms as well as suggest treatments tailored for individual patients. It is important to expand these studies to a larger cohort of patients with detailed analysis of their biochemical parameters and clinical phenotypes to determine the robustness and stability of the various subtypes and their connection with genetic polymorphisms and other genetic parameters. We expect that similar use of microarrays and advanced bioinformatics will greatly enhance our understanding of pathophysiological mechanisms in various erythroid diseases involving abnormal genetic regulation of erythropoiesis (i.e., aplastic anemia or polycythemia vera), as well as erythrocyte-related dysfunction or hemoglobinopathies (i.e., hemolytic anemia, sickle cell disease).

Without novel mRNA synthesis, post-transcriptional regulation is probably the main mechanism for gene regulation in enucleated cells (reticulocytes, platelets, and erythrocytes). Given the programmed transcriptional arrest with nuclear exclusion in developing erythroid cells, significant gaps sometimes exist between the time of mRNA synthesis and actual translation. RNA stability and translation control are therefore tightly regulated to ensure proper control of RNA stability and protein synthesis [43], both of which are likely to be regulated by microRNAs. For example, the post-transcriptional control of CD71 is mediated by the actions of iron regulatory proteins (IRP1 and IRP2) in response to iron levels [21]. The loss of surface expression of CD71 during erythrocyte terminal differentiation was thought to be caused by its release/secretion by exosomes [44]. Our findings suggest that microRNAs are also involved in the reticulocyte terminal differentiation process. Since miR-320 expression does not change during the transition between reticulocyte and erythrocytes, its upregulation is not likely to be a trigger for the loss of CD71 expression. Instead, miR-320 is likely to fine tune the translational activities of CD71 in reticulocytes and contribute to its loss of CD71 surface expression together with the exosome release. MicroRNA-mediated repression can also be regulated by interactions between miRNA/Argonaute complexes with RNA-binding proteins that relocate from different subcellular compartments during stress or differentiation [45]. Many additional mRNAs encode proteins with important regulatory roles in erythropoiesis are also predicted targets of erythrocyte microRNAs [24] and their interactions deserve further exploration in the context of various physiological and pathological adaptations of erythrocyte diseases.

## Materials and Methods

### Human blood collection, mature erythrocyte purification and cell lines

This study was conducted with the approval of the Duke University Institutional Review Board (IRB), and informed consent from each donor was obtained. Blood was obtained by venipuncture and collected into sodium citrate-containing tubes. Leukocytes were first removed by a PALL PurecellNeo filter (Pall Biomedical Co, NY). The filtrate was separated in a Ficoll-hypaque gradient to eliminate remaining leukocytes and platelets and further leukocyte and reticulocyte depletion using antibody-conjugated magnetic beads (conjugated to CD45 [leukocyte common antigen, LCA] and CD71 [transferrin receptor] antibodies (Miltenyi Biotec, CA). Alternatively, whole blood was subjected to Ficoll-hypaque gradient centrifugation to remove platelets and leukocytes. Packed erythrocytes were then subjected to arabinogalactan density gradient (Larex, MN), comprising three layers—1.085, 1.090, and 1.095 g/ml, to separate reticulocytes from mature erythrocytes, which migrated to the high density layer and were collected for RNA extraction. The purity of the resulting erythrocytes was evaluated with FACS for surface expression of CD235a (glycophorin A), CD71 (transferrin receptor), CD16 (FcγRIII) and CD45 (LCA) with fluorescently labeled antibodies (Pharmingen, CA). To obtain reticulocytes used for terminal differentiation assays and gene expression studies, packed erythrocytes obtained from leukocyte-removal filtrate were incubated with CD71-MACS beads (Miltenyi). After washing with staining buffer (Hanks' balanced salt solution with 2% FCS and 0.02% NaN3), CD71+ reticulocytes were enriched with autoMACS by POSSEL_S mode, immunostained with FITC-conjugated CD71 antibodies and positive cells sorted by FACS. The K562 cells (from Dr. Murat Arcasoy) were maintained in RPMI supplemented with 10% fetal calf serum, glutamine and antibiotics.

#### RNA extraction, electrophoresis, RT-PCR analysis, and Northern blot analysis

Total RNA was extracted using the mirVana microRNA Isolation Kit (Ambion, TX). For northern blot analyses, 20 µg of total RNA were separated in 15% TBE-urea polyacrylamide gel (Bio-Rad, CA), and electro-transferred onto Hybond-N+ (Amersham Biosciences, UK) membranes. The blot was then probed with end-labeled DNA probes complementary to each indicated microRNAs or U6 RNA in Express-hyb buffer (Clontech, CA) at 37°C.

### Analysis of microRNA expression by microarrays

For microRNA expression profiling by microarray, total RNA samples were size-fractionated and cleaned with the flashPAGE Fractionator (Ambion, TX) to collect RNAs smaller than 40 nucleotides. These samples then were labeled with the mirVana microRNA Labeling Kit (Ambion) according to the manufacture's instructions [46]. Erythrocyte and K562 small RNAs were labeled with Cy5; while 293T small RNAs were labeled with Cy3 as common reference for all samples. The fluorescently labeled microRNAs were mixed with 3× microRNA Hybridization Buffer (Ambion) and heated at 95°C for 3 min before hybridizing with the printed microRNA microarrays for 12–16 h in a 42°C water bath in sealed cassettes. Following hybridization, the slides were washed, dried and scanned on a GenePix 4000B Array Scanner (Axon, CA). Array images were analyzed using the GenePix Pro 5.0 software (Axon) and submitted into the Duke Microarray Database (DMD). Data were normalized globally per array, such that the average LogRatio was 0 after normalization. All data used for analysis had an average intensity 2.5 fold above local background, and a regression ratio >0.6 before further filtering based on indicated variations among different samples. Hierarchical clustering was performed by average linkage using uncentered Pearson correlation [47]. Microarray data has been submitted into GEO database with accession number GSE11060 and available on the supplemental website (URL: http://data.genome.duke.edu/RBC).

### Expression profiling of microRNA using multiplexing RT-PCR assays

The 192-plex RT-PCR assays that were developed for profiling of 192 individual human microRNAs were used to perform microRNA profiling with 30 ng of total RNA from each RBC/reticulocyte samples following the three-step protocols described previously [17]. Briefly, step 1 is a multiplexed reverse transcription reaction which reverse transcribes targeted microRNAs into cDNAs in a single reaction using 192 microRNA-specific stem-loop RT primers; step 2 is a multiplexed PCR reaction with 192 sets of microRNA-specific forward primers and a universal reverse primer that amplifies the cDNA products to provide enough samples for step 3. Linear amplification was achieved with 14 PCR cycles. Step 3 is done as simultaneous, individual single-plex TaqMan® real-time PCR reactions to monitor the abundance of each microRNAs after the multiplexed RT-PCRs. Real-time PCR was performed on an AB 7900 HT Sequence Detection System in a 384-well plate format, with the temperature regime consisting of a hot start at 95°C for 10 min, followed by 40 amplification cycles composed of 95°C for 15 s, and 60°C for 1 min. The real-time PCRs for each microRNAs were run in duplicates. Out of 192 microRNA targets, only 189 assays lead to expression information since three assays (miR-16, -124a and -130b) had technical issues and were not included in the analysis. MiR-152 was chosen as the endogenous control for normalization across different samples based on two reasons: 1) the level of miR-152 does not change during erythroid differentiation [14]; 2) has-miR-152 showed the least standard deviation (Stdev = 0.68) across 40 tissues in a global microRNA profiling study using 210 microRNA TaqMan assays (Applied Biosystems, unpublished data). A −ΔCt ( = Ct_microRNA−Ct_miR-152, Ct: cycle of threshold) was calculated as an equivalent of normalized relative gene expression level for the microRNAs being analyzed and used for hierarchical clustering.

### In vitro maturation assays of reticulocytes and miR-320 knockdown

The reticulocyte *in vitro* maturation assay was described previously [22]. The CD71+ young reticulocytes were isolated from leukodepletion-filtered erythrocytes with CD71 microbeads using autoMACS^tm^ Separator. Flow cytometry and new Methylene Blue staining was performed to determine the purity of the isolated cells. The purified reticulocytes were then cultured in RPMI supplemented with 10% fetal bovine serum and 50% autologous plasma in a humidified atmosphere of containing 5% CO2. Locked Nucleic Acid (LNA) oligonucleotides against miR-320, mir-20a and scrambled control sequence (Exiqon, Denmark) were individually diluted in Opti-MEM and mixed with diluted Lipofectamine 2000 (Invitrogen) for 30 min at room temperature before transfecting into reticulocytes at a final concentration of 33 nM. Transfection medium was replaced 6 hours post-transfection with the differentiation media composed of RPMI containing 10% FCS and 50% autologous plasma. CD71 expression levels of transfected cells were analyzed by FACS at indicated time after transfection.

### Cloning of CD71 reporter constructs and luciferase assays

The 3′UTR of the CD71 was amplified from a K562 cDNA with a pair of primers (forward: atgtgatacccatagcttcc; reverse: ggcttagatctcatttggag) and cloned into the XbaI site downstream of firefly luciferase gene in the pGL3-control (Promega, Wisconsin). The CD71 3′UTR miR-320 mutant reporters were constructed with QuikChange® II Site-Directed Mutagenesis Kits (Stratagene, CA), which created three base pair changes in the miR-320 seed sequence-targeted regions (underlined) (CTAGATGTCTTTAGGCAG***G***A***T***C***C***TTTAA to replace CTAGATGTCTTTAGGCAGCAGCTTTTAA). To assess the effect of miR-320 on CD71 3′UTR activity, expression constructs encoding miR-320 and miR-451 were inserted into a CMV-based pcDNA3 cloning vector (Invitrogen, CA). The following primers were used to amplified the expression constructs from the genomic DNA of K562 cells and cloned into the XhoI and EcoRI site of pcDNA3: miR-320 (forward: ccgaattccaggaaccagacagggacgc; reverse: ccctcgagccgactcttaagtccaggtc) and miR-451 (forward: ccgaattcacagtgcttttcaagccatgc; reverse: ccctcgagatcctcctgccttggcctctg). The functions of these expression constructs have been confirmed by their ability to specifically repress pGL3 luciferase “sensor” constructs comprising two copies of the perfect matched sequences. K562 cells were cotransfected with 1 µg of indicated reporter (CD71 3′UTR or mutCD71 3′UTR), 50 ng renilla luciferase construct and 2 µg of indicated microRNA expression constructs, all combined with Lipofectamine 2000 (Invitrogen). After 48 hour, the transfected cells were washed and lysed with the passive lysis buffer (Promega). The luciferase activities (both firefly and renilla luciferase) present in the cell lysates were then determined by Luminometer (Berthold Technology, Germany). The relative reporter activities were obtained by normalization of firefly to the renilla luciferase activities determined in the same lysates with the Dual-Glo Luciferase Assay (Promega).

## Supporting Information

Figure S1 The percentage change of indicated parameters (MHC, MCHC) compared with the corresponding parameter at time zero during the process of terminal differentiation *in vitro*.
(0.76 MB EPS)

Table S1 The mRNA targets for miR-320 predicted by TargetScanS.
(0.33 MB XLS)

Table S2 The mRNA targets for miR-320 predicted by PicTar
(0.62 MB XLS)
